# KLu(WO_4_)_2_/SiO_2_ Tapered Waveguide Platform for Sensing Applications

**DOI:** 10.3390/mi10070454

**Published:** 2019-07-05

**Authors:** Marc Medina, Christian E. Rüter, Maria Cinta Pujol, Detlef Kip, Jaume Masons, Airán Ródenas, Magdalena Aguiló, Francisco Díaz

**Affiliations:** 1 Física i Cristallografia de Materials i Nanomaterials (FiCMA-FiCNA-EMaS), Departament de Química Física i Inorganica (DQFI), Universitat Rovira i Virgili (URV), Campus Sescelades, C/Marcellí Domingo 1, E-43007 Tarragona, Spain; 2 Faculty of Electrical Engineering, Helmut Schmidt University, 22043 Hamburg, Germany; 3 Departamento de Física, Instituto de Estudios Avanzados (IUdEA), Universidad de la Laguna (ULL), E-38200 San Cristóbal de La Laguna, Spain

**Keywords:** tapered waveguides, ultraprecise dicing saw, biochemical sensors, label-free

## Abstract

This paper provides a generic way to fabricate a high-index contrast tapered waveguide platform based on dielectric crystal bonded on glass for sensing applications. As a specific example, KLu(WO_4_)_2_ crystal on a glass platform is made by means of a three-technique combination. The methodology used is on-chip bonding, taper cutting with an ultra-precise dicing saw machine and inductively coupled plasma-reactive ion etching (ICP-RIE) as a post-processing step. The high quality tapered waveguides obtained show low surface roughness (25 nm at the top of the taper region), exhibiting propagation losses estimated to be about 3 dB/cm at 3.5 μm wavelength. A proof-of-concept with crystal-on-glass tapered waveguides was realized and used for chemical sensing.

## 1. Introduction

Optical sensors such as flow cytometers and microplate readers have long been used to analyze biological and chemical samples. New devices have emerged in the past decade, due to optical bulk sensor miniaturization, which has evolved to integrated optical microsystems such as on-chip waveguides and resonators. There exists an extensive and wide bibliography about integrated optics and optofluidics for sensing purposes. These sensors can be applied or potentially applied in fields such as health diagnosis, environmental monitoring, food industry and veterinary, industrial process control, and pharmaceuticals, among others [1,2,3,4,5,6].

Some miniaturized sensors using refractive index (RI) detection, surface-enhanced Raman spectroscopy (SERS) and fluorescence detection have already been fabricated and tested [6]. For RI detection some architectures including optical microcavities [7], antiresonant reflecting optical waveguides (ARROWs) [8,9], optical ring resonators [10,11,12] and photonic crystals [13,14], have already been explored. Most of the aforementioned sensors are operating in the visible wavelength range, nonetheless some studies have been done at the near-infrared (NIR) wavelength region—in waveguide configuration for glucose determination [15,16]; a gas sensor in microarray configuration [17]; another gas sensor on Si waveguide architecture [18]; a Si on BTO chemical sensor-on-chip [19]—have obtained satisfactory and promising results [20].

The tapering in waveguides has been extensively used in tapered optical fiber sensing [21,22,23]. As Korposh et al. [21] claimed, the main advantage of the tapering is that it provides large access to the evanescent wave of the propagating mode, so it increases the interaction between the guided light and the surrounding medium, allowing the measurement of its chemical nature. In the same review, it is claimed that single taper-based RI sensors acting in the RI range 1.36–1.46 show a sensitivity of about 10^−4^ RIU. In addition to tapered fibers, some taper geometry configurations have been explored with tapered polymer waveguides [24,25] and a SiN on MgF_2_ tapered waveguide [26]. Even some experiments on Nd-doped waveguides for lasing with tapered geometry have already been proved [27].

The present work proposes the fabrication of evanescent-field enhanced waveguides as a signal transducer and a high throughput generic process for sensor chip fabrication based on a top-down approach. In a first study, a high-index waveguide configuration is used with KLu(WO_4_)_2_ (henceforth abbreviated as KLuW) [28] as a core and SiO_2_ as a mechanical support and cladding compound. KLuW is chosen based on the research group extensive experience in using this material. KLuW also provides a large refractive index contrast with SiO_2_ of around 0.4, and the possibility to operate at the chemical and biological fingerprint region due to its broad transmission window from visible up to 5.5 µm wavelength. This partial transparency at the fingerprint region, which is located at the mid-infrared (MIR) zone and is known because it contains many molecular groups characteristic and specific absorption structures, makes KLuW an attractive material for chemical sensing based on substance molecular groups. Moreover, KLuW crystals possess high absorption and emission cross-sections when doped with active lanthanide ions, and have the capability to be doped with high concentrations of active ions for in-situ laser light generation. These large absorption and emission cross sections could be relevant if the KLuW crystals are doped with active ions, and their optical spectroscopic features are used as a base for the signal used in chemical sensing. In previous studies, KRE(WO_4_)_2_ (being RE = Sc,Y and Ln, all these compounds are isostructural) on SiO_2_ systems were evaluated as platforms for high refractive index waveguide platforms, but treated at simulation level [29,30]. An experimental demonstration by Sefunc et al. [31] obtained 200–300 micrometers-length rib waveguides fabricated by Focused Ion Beam (FIB) technology.

The applied generic recipe for chip fabrication is based on the combination of three techniques: chip bonding, taper cut with ultraprecise dicing saw machine [32,33,34,35] and inductively coupled plasma reactive ion etching (ICP-RIE) as a post-processing step.

This work presents the first phase of the project, which consists of the design and fabrication of tapered waveguides in KLuW on silica for sensing applications, as well as its structural and optical characterization, followed by a chemical sensor proof of concept with ethanol. In the second phase of the project, it is planned to delve into the theoretical calculations related to the design and optimization of the tapered waveguides, and in addition and most important, the realization of detailed measurements of the waveguides performance for bio/chemical sensing.

## 2. Materials and Methods

A tapered waveguide geometry was chosen because it guarantees the sensor implementation on a chip by allowing a low-loss direct interconnection with embedded commercial fibers for light coupling and signal collection. It also allows for later construction of microfluidic channels as a sample delivery system. Therefore, the presented sensor belongs to the direct input/output (DIO) sensors category where sensing relies on the interaction between the evanescent wave of the exposed waveguide surface and the sample of interest.

The sensor design aims to fulfill general requirements for the construction of an optimal optical sensor. The integration of all components is the major characteristic. For this reason, the final design has to be compact. Such sensors guarantee high sensitivity and are able to perform real-time and label-free on-site measurements. In the case of the present research, a tapered waveguide configuration was chosen due to its capability to be integrated and do multiplexing.

There are two main objectives in the design of tapered waveguides. In the first place, the waveguides require that they could be implemented on a sensor chip integrating optical fibers for light coupling and signal collection and microfluidics as a sample delivery system. The second requirement is that they guarantee the light coupling to the waveguides without further technological effort. Therefore, they are designed to overcome the common difficulties existing in other waveguide designs. Herein, a tapered waveguide configuration is proposed. It assures a good fiber coupling at the input and output of the waveguides, and the waveguide is thin enough in the central region to possess high sensitivity for sensing.

In order to allow for the fabrication of tapered waveguides, a heterogeneous system was chosen. By bonding KLuW crystal plates to SiO_2_ (amorphous glass) the system will reach about 0.4 index contrast, which enables the fabrication of low loss waveguides. Furthermore, waveguide dimensions will also depend on the wavelength at which the sensor will operate. This study focuses on the MIR mainly because working in this wavelength range would increase the evanescent field penetration depth and would allow the sensor to identify species by its MIR fingerprints.

**Figure 1:**
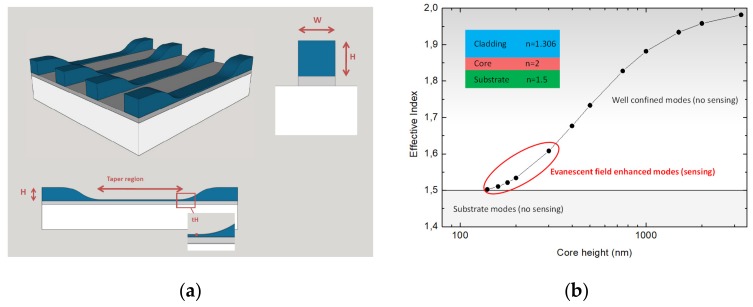
(**a**) Tapered waveguide design scheme, being W the width of the waveguide, H the height of the un-tapered region. The zoom region shows the tH the thickness of the taper region. (**b**) Calculation of the waveguide mode dispersion as a function of the height of the ridge waveguide, the inset shows the refractive indexes used for each layer for the calculation.

To choose initial dimensions for the tapered design, all possible options below one micrometer thickness (by 100 nm steps) were simulated by using COMSOL Multiphysics^®^ software (COMSOL Inc, Burlington, MA, USA), choosing the optimal ones among all possibilities. Results are shown as a graphic in Figure 1b. The target height dimension for the taper region is below 300 nm.

## 3. Results

### 3.1. Sensor Fabrication and Characterization (Top-Down Approach)

The most challenging part of this study was to fabricate taper-shaped waveguides. Most common microfabrication techniques such as photolithography followed by wet or physical etching steps did not allow accurate control on the vertical dimension (normal to flat sample plane) to transfer the taper structure, i.e., the small slope necessary to guarantee low-loss waveguides at the start and end of the taper region in the waveguide. One important limitation of common microfabrication techniques is that when they transfer a design to the sample by etching in the direction normal to the sample plane, this etching is homogeneous in height in the whole sample. The most popular approach to overcome this difficulty would be soft lithography, which permits translating complex 3D geometries to a polymer mask. Then a subsequent etching step would make it possible to shape the sample with a taper geometry. Although this technique would satisfy the established requirements, regarding 3D geometry there is a limitation with etching procedures for KLuW. This material is inert for all wet chemical etchants showing a very low etch rate and anisotropic etching with hydrofluoric acid. Those characteristics make it difficult to etch KLuW with most of the wet and dry (physical and chemical) etching techniques.

Bearing in mind the taper configuration and the difficulty of etching KLuW, an ultra-precise dicing saw machine was chosen to fabricate tapered waveguides. Dicing saws are commonly used in microchip fabrication to cut silicon wafers into pieces. They provide enough spatial resolution in all three dimensions to fabricate the designed waveguides including the taper shape, and further on the complete set of waveguides for final device fabrication. The dicing saw machines’ capability to mill KLuW exclusively depends on the blade composition and characteristics, and there is a huge range of different grids and base materials for dicing blades commercially available. Other advantages of this technique used to fabricate waveguides are its high speed, high performance, and easy use. In addition, it is flexible and versatile (both in changing to new designs), and it can work on different materials. It also allows for the manufacturing of high aspect ratio waveguides and finally it is scalable to an industrial level. As a major limitation, it is unable to cut curves in the plane of the waveguide, since the saw is only capable of cutting straight lines.

**Figure 2:**
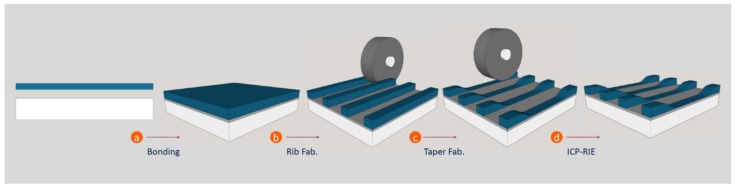
Taper waveguide fabrication scheme. (**a**) KLuW/NOA83H/SiO_2_ bonding step (NOA stands for Norland Optical Adhesive). (**b**) Rib waveguide fabrication. (**c**) Taper waveguide cut. (**d**) Inductively coupled plasma reactive ion etching (ICP-RIE) post-processing.

Finally, a combination of three techniques was used for the fabrication of tapered waveguides: chip bonding, taper cut with an ultra-precise dicing saw machine and ICP-RIE. The final ICP-RIE step was carried out in order to assure the final thickness of the waveguide in the sensing region to be thin enough in order to enable a strong evanescent field. Figure 2 summarizes the taper waveguide fabrication.

To meet the specifications from Section 2 it was decided to fabricate an initial set of 20 taper waveguides, equidistant at 100 µm and with around 20 × 20 µm in horizontal and vertical dimensions. Crystalline KLuW plates were obtained from bulk KLuW crystals grown at our crystal growth laboratory. The crystal growth was carried out by a high-temperature solution method using the top seeded solution growth by slow cooling technique. A more detailed description can be found in previous literature [13]. The single crystals obtained are defect-free and they belong to the monoclinic crystal system. After the crystal growth procedure, the crystals are cut and polished with a Logitech PM-5 polishing system. The thinning process consists of a succession of scalable steps where the sample thickness is reduced by using three different abrasive colloidal dispersions, starting with 9 µm diameter alumina grain size, followed by 3 µm and 1 µm alumina grain size. A final fine polishing step was performed with SF1 from Logitech (an alkaline colloidal silica suspension) in order to obtain an optical quality surface with <5 nm (rms) roughness. The plates’ final thickness obtained was 1 mm, with <4 nm (rms) surface roughness. Fused silica glass, commercial Suprasil 300 from Heraeus, samples are plates of 5 × 5 cm, and 1 mm thick. They were cut with the dicing saw to 2 × 2 cm plates to make them more manageable.

Chip bonding starts with a cleaning step to assure that both glass and KLuW surfaces are clean enough to ensure high quality and long-lasting bonding. First, both samples are cleaned by dipping them in acetone and subsequently in isopropanol. After that, both samples are cleaned in a RIE chamber by means of an oxygen plasma (550 W, 90 mTorr, 100 sccm, 300 s). After cleaning NOA83H is spin-coated (500 rpm for 10 s, 1500 rpm for 40 s) on KLuW or glass (NOA stands for Norland Optical Adhesive). Just after the spin-coating, both samples are sticked together with no pressure and placed in a vacuum chamber at 9·10^−3^ mTorr for 5 min in order to remove any possible bubble that can be trapped at the interface. Then, NOA adhesive is cured with 265 W UV lamp (in the case of NOA83H at its optimum dose of 8.5 mW/cm^2^) for 280 s. Finally, to do a subsequent heating step is necessary to guarantee NOA83H is fully cured (in this case a 30 min ramp until 358 K staying at this temperature for 4 h and then cooling down at 1 K/min). The glue interface showed high quality without bubbles or defects.

Tapered waveguides were fabricated with an ultra-precise dicing saw Disco DAD322. To fabricate 20 × 20 µm waveguides the first step consists of chopping the KLuW layer down to a thickness close to 40 µm. Then the surface is polished down to 20 μm with a softer blade. Thereafter, the disc blade is dipped into the KLuW to fabricate the tapered structure. Finally, straight channels are cut by using a 100 µm thick resin-bonded blade at 20,000 rpm and a cutting speed of 0.1 mm/s. Similarly, the end faces were prepared by making a straight cut perpendicular to the waveguides at both edges.

After dicing fabrication, waveguides thickness is still thicker than desired; hence, it is necessary to post-process the sample. ICP-RIE is a very reasonable technique to do this. To avoid the breakage of the thin sample we used a multi-step approach. For each etching step the following parameters were used: 12 mTorr pressure, 200 W RF/350 W ICP (395 V), 10 sccm Ar: 10 sccm SF_6_ gas flow, duration 120 sec, followed by an Ar purge step at 20 mTorr pressure, 10 sccm Ar gas flow, for 300 sec. This treatment was repeated 30 times leading to a total etching time of 2 h 30 min.

**Figure 3:**
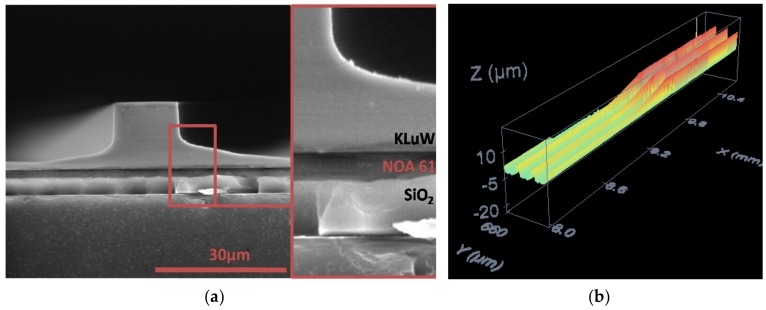
(**a**) Environmental scanning electron microscope (ESEM) image of the cross section of the waveguide platform. (**b**) Confocal microscope topography of tapered waveguides.

Tapered waveguides show high quality; they have a low surface roughness (25 nm rms roughness, measured with a confocal microscope) and no chipping at waveguides edges. Therefore, regarding height, the dicing saw technique is accurate enough to get high-quality taper waveguides. Figure 3a shows an environmental scanning electron microscope (ESEM) top image of two tapered waveguides after dicing saw fabrication. In Figure 3b a topographic image, obtained using a confocal microscope of three tapered waveguides is presented.

**Figure 4:**
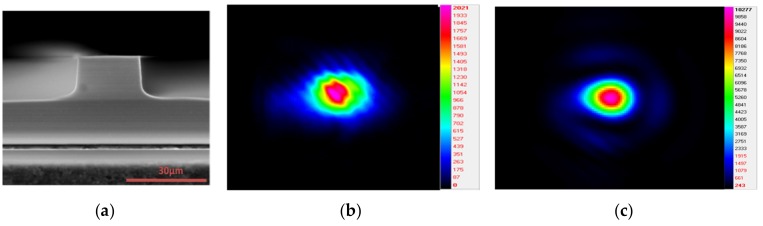
(**a**) ESEM cross section of a diced ridge waveguide. (**b**,**c**) show the near field distribution of Gaussian input and output beam (3.68 µm) coupled and that obtained from the end face of the tapered waveguide, respectively.

The waveguides were optically characterized by coupling 3.68 µm coherent light directly to the tapered waveguide by means of an optical lens. Near field distribution from the output face was collected by a lens and then captured by an infrared (IR) camera. The resulting experimental information was used to estimate propagation losses, which are around 3 dB/cm. This value should not be strictly considered as propagation losses because of the non-constant core height along the waveguide. However, it can be considered as a guide used to track the increase in propagation losses as a consequence of RIE steps. Figure 4a shows an ESEM cross-section picture of one of the tapered waveguides before ICP-RIE post-processing. The end face shows high quality, and blade polishing has turned out to be a nice approach for end face preparation. Figure 4b corresponds to the near field distribution of the Gaussian input beam (at 3.68 µm), coupled to the tapered waveguide. It was not possible to determine experimentally if the taper waveguides are monomode or multimode. Figure 4c shows the near field distribution of the Gaussian output beam (at 3.68 µm) obtained from the tapered waveguide (Figure 4a) end face.

### 3.2. Sensor Proof of Concept

The chemical sensor prototype proof of concept was carried out by using static analytic solution drops as a chemical detection target. These drops were directly released at the waveguide taper region by using a micropipette. An MIR laser diode (ICL Nanopulse) emitting at 3.68 µm was selected as a light source working at vertical polarization (transversal magnetic, TM) and directly coupled to the waveguide by a lens.

After RIE postprocessing, tapered waveguide with final dimensions of 9 µm in height and 20 µm in width (H = 9 µm and W = 20 µm) and the thickness tH in the taper region of around 800 nm are selected, measured with the confocal microscope. All spectral measurements were normalized to an air reference previously measured at the same nominal temperature, minimizing potential chromatic effects associated with the whole optomechanical and photonic system.

Water, ethanol and mixtures of both constituents (in different proportions) were the chemical products employed, using water as a solvent. In this experiment, 10 microliters were selected as a droplet volume for each of the solutions measured. The applied droplets covered around 7 mm of the studied waveguide (in which the tapered zone has a total length of 8 mm).

**Figure 5:**
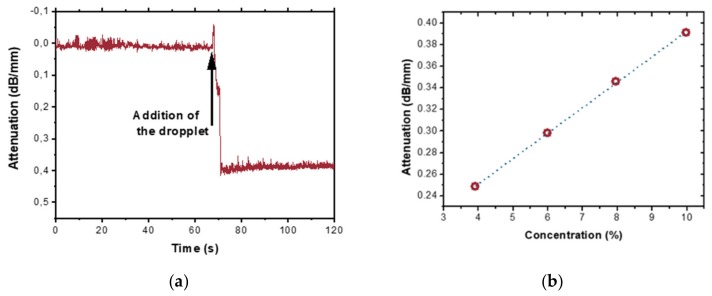
Sensing demonstration (**a**) Example of a sensor experiment where a droplet of 10 wt.% ethanol/water solution has been applied to the tapered section of the waveguide. (**b**) TM-polarized light absorption induced by the water/ethanol solution droplets on the sensor surface at λ = 3.68 μm wavelength as a function of ethanol concentration.

In the example given in Figure 5a, 10% ethanol/water solution gave a signal attenuation of 0.4 dB/mm, i.e., close to 40% of total output intensity signal was absorbed. Figure 5b demonstrate tapered waveguides capability for sensing, showing a linear response for ethanol–water dissolutions ranging from 4 to 10 vol.%. Those two figures are taken as a proof of concept for tapered waveguides sensor potential.

## 4. Conclusions

KLuW crystal plates were successfully bonded to silica by using NOA83H. The bonding interface shows a high quality with no defects or bubbles. The adhesive bonding layer shows negligible effects on the optical characteristic of the tapered waveguides (such as absorption). Tapered KLuW-on-glass waveguides were successfully fabricated by using ultra-precise dicing saw (DISCO DAD332). Dicing produces waveguides with low roughness (25 nm rms roughness at the top of tapered waveguides). The dicing saw is also able to produce a smooth taper transition. The reactive ion etching post-treatment kept the taper geometry (there was no substantial modification in the taper geometry), and therefore it can be successfully used to reduce the thickness of the KLuW waveguides. Waveguide experiments with 3.68 µm laser were favorably undertaken, with reasonable losses around 3 dB/cm. Tapered waveguides proof of concept for sensing demonstrates that KLuW/SiO_2_ tapered waveguides configuration can potentially be used as a chemical sensor.

Despite the good results, tapered waveguides require further optimization in terms of fabrication. It is also necessary to carry out extensive studies on the design to further improve the waveguide performance. Although it has been demonstrated that these guides can be used as sensors, there is still a long way to go before they can be compared in terms of performance with those of the bibliography, since the optimal conditions have not yet been achieved.
